# *Hmga2* deficiency is associated with allometric growth retardation, infertility, and behavioral abnormalities in mice

**DOI:** 10.1093/g3journal/jkab417

**Published:** 2021-12-08

**Authors:** Mi Ok Lee, Jingyi Li, Brian W Davis, Srijana Upadhyay, Hadil M Al Muhisen, Larry J Suva, Tracy M Clement, Leif Andersson

**Affiliations:** 1 Department of Veterinary Integrative Biosciences, Texas A&M University, College Station, TX 77843, USA; 2 Department of Veterinary Physiology and Pharmacology, Texas A&M University, College Station, TX 77843, USA; 3 Interdisciplinary Program in Toxicology, College of Veterinary Medicine and Biomedical Sciences, Texas A&M University, College Station, TX 77843, USA; 4 Department of Medical Biochemistry and Microbiology, Uppsala University, SE-75123 Uppsala, Sweden; 5 Department of Animal Breeding and Genetics, Swedish University of Agricultural Sciences, SE-75007 Uppsala, Sweden

**Keywords:** high mobility group AT-hook 2, CRISPR/Cas9, sterility, behavior

## Abstract

The high mobility group AT-hook 2 (HMGA2) protein works as an architectural regulator by binding AT-rich DNA sequences to induce conformational changes affecting transcription. Genomic deletions disrupting *HMGA2* coding sequences and flanking noncoding sequences cause dwarfism in mice and rabbits. Here, CRISPR/Cas9 was used in mice to generate an *Hmga2* null allele that specifically disrupts only the coding sequence. The loss of one or both alleles of *Hmga2* resulted in reduced body size of 20% and 60%, respectively, compared to wild-type littermates as well as an allometric reduction in skull length in *Hmga2*^−/−^ mice. Both male and female *Hmga2*^−/−^ mice are infertile, whereas *Hmga2*^+/−^ mice are fertile. Examination of reproductive tissues of *Hmga2*^−/−^ males revealed a significantly reduced size of testis, epididymis, and seminal vesicle compared to controls, and 70% of knock-out males showed externalized penis, but no cryptorchidism was observed. Sperm analyses revealed severe oligospermia in mutant males and slightly decreased sperm viability, increased DNA damage but normal sperm chromatin compaction. Testis histology surprisingly revealed a normal seminiferous epithelium, despite the significant reduction in testis size. In addition, *Hmga2*^−/−^ mice showed a significantly reduced exploratory behavior. In summary, the phenotypic effects in mouse using targeted mutagenesis confirmed that *Hmga2* is affecting prenatal and postnatal growth regulation, male reproductive tissue development, and presents the first indication that *Hmga2* function is required for normal mouse behavior. No specific effect, despite an allometric reduction, on craniofacial development was noted in contrast to previous reports of an altered craniofacial development in mice and rabbits carrying deletions of both coding and noncoding sequences at the 5′ part of *Hmga2*.

## Introduction

High mobility group AT-hook 2 (HMGA2) belongs to the nonhistone chromosomal high mobility group (HMG) protein family and is well-conserved in mammals (Ozturk *et al.* 2014; Colombo *et al.* 2017). As an architectural transcription factor, both the level of *HMGA2* expression and biochemical modifications of the HMGA2 protein affect transcriptional regulation of downstream targets of various signal transduction pathways (Singh *et al.* 2015; Dobersch *et al.* 2021). HMGA2 preferentially binds to AT-rich DNA regions in the genome, interacts with a large number of proteins, mostly transcription factors, and regulates the expression of numerous genes thus affecting diverse biological processes including growth, cell proliferation, differentiation, and death, as well as tumor development (Singh *et al.* 2014, 2015; Colombo *et al.* 2017).

In mice, the first three exons of *Hmga2* encode the AT-hook structural DNA-binding domain that binds specifically to the minor groove of AT-rich sequences (Colombo *et al.* 2017). This interaction allows the formation of a transcriptional complex proposed to act as a transcriptional regulating factor for either promoting or inhibiting transcription (Colombo *et al.* 2017). The remaining two exons encode an acidic carboxyl terminus with as yet unknown function (Singh *et al.* 2015; Colombo *et al.* 2017). *Hmga2* is highly expressed during embryonic development and in rapidly proliferating cells, whereas the expression is low to absent in most adult tissues except the testes (Zhou *et al.* 1995; Hirning-Folz *et al.* 1998; Liu *et al.* 2000; Chieffi *et al.* 2002). In humans, *HMGA2* is classified as an “oncofetal” gene that promotes tumor progression and metastasis when overexpressed in cells (Cattaruzzi *et al.* 2007). A chromosomal rearrangement involving *HMGA2* is frequently detected in many human cancers (Ashar *et al.* 1995; Baldassarre *et al.* 2001; Singh *et al.* 2015).

Evidence for the specific role of HMGA2 *in vivo* comes from multiple naturally occurring or induced mutations in mammals. The identification of a 12q14 microdeletion around *HMGA2* in patients with short-stature supports an important role for this locus in controlling human growth (Lynch *et al.* 2011; Alyaqoub *et al*. 2012). Human GWAS (genome-wide association study) have revealed that *HMGA2* is one of the loci most consistently associated with stature across human populations (Weedon *et al.* 2007). In addition, naturally occurring mutations, including deletions of the 5′ region of *Hmga2* causing the pygmy phenotype in mice (King 1950) and dwarfism with altered craniofacial development in rabbits (Carneiro *et al.* 2017), confirm an important conservation of function for HMGA2. *HMGA2* is also known to be associated with variations in body size in chicken (Song *et al.* 2011), dog (Jones *et al.* 2008; Zapata *et al.* 2016), horse (Makvandi-Nejad *et al.* 2012; Kader *et al.* 2015), and pig (Chung *et al.* 2018), as well as with beak size in Darwin’s finches (Lamichhaney *et al.* 2016) and height in cattle (Bouwman *et al.* 2018). Thus, *HMGA2* is one of the most important loci regulating body size in vertebrates.

Four different *Hmga2* murine knockout models have previously been characterized (King 1950; Benson and Chada 1994; Zhou *et al.* 1995; Chieffi *et al.* 2002; Chiou *et al.* 2014). The spontaneous *pygmy* mutation (*Hmga2^pg^*) constitutes a deletion of the first two exons and several kb upstream of *Hmga2*, and was reported to be associated with both reduced body size and altered craniofacial development (King 1950). Male *Hmga2^pg^* homozygotes develop priapism (prolonged erection of the penis) and both sexes are sterile (King 1950). A transgenic insertional mutant *Hmga2^pg-TgS40ACha^* showed a similar phenotype as *Hmga2^pg^* mice with small body size and disproportionately larger brain than other organs (Benson and Chada 1994). A chromosomal inversion *In(10)17Rk* resulting in a fused *Hmga2* transcript with a novel 5′ sequence in which *Hmga2* exon 4 and 5 combined with *Txlnb* (taxilin beta) exon 1 and exon 2, also caused a similar pygmy phenotype including reduced birth weight and brachycephaly (Zhou *et al.* 1995). Later, undescended testis with abnormal spermatogenesis was reported in *In(10)17Rk* male homozygotes (Chieffi *et al.* 2002). Taken together, the preponderance of genetic evidence supports an evolutionary well-conserved role for *Hmga2* and that a variety of null mutations are consistently associated with small body size. We noted, however, that these naturally occurring and targeted null mutations, involve deletions of both coding and noncoding regions, and in some cases deletions of large genomic regions (King 1950; Benson and Chada 1994; Zhou *et al.* 1995). Therefore, these studies do not exclude the possibility that the reported phenotypic effects may be the result of disruption of regulatory elements and not specifically due to HMGA2 function. However, *Hmga2^GFP^* mice which induced conditional inactivation of exon 2 and a GFP fusion supports a direct effect of HMGA2 in controlling body size, but the phenotypic characterization of this mouse model was restricted to only weight and height (Chiou *et al.* 2014).

In this study, a loss-of-function *Hmga2* mouse model was generated using CRISPR/Cas9 to disrupt the coding sequence without affecting adjacent noncoding sequences. The subsequent phenotyping analyses explored whether HMGA2 function directly contributes to body size, fertility, and craniofacial development defects. Using this new mouse model, the specific role of *Hmga2* in controlling body and organ size and fertility was confirmed, a possible effect on behavior was noted but no specific effect on craniofacial development was detected.

## Materials and methods

### Ethical statement and animal care

This study followed institutional guidelines for the care and use of laboratory animals. The Institutional Animal Care and Use Committee (IACUC) of Texas A&M University approved this study (animal use protocol number 2018-0166). Animals were housed in polypropylene cages with standard pellet diet and water *ad libitum*. Room temperature was maintained at 22°C ± 2°C and a relative humidity of 60% ± 5% on a 12-h light/dark cycle.

### Generation of *Hmga2^tamu-ko^* mice using CRISPR-Cas9

Texas A&M Institute for Genomic Medicine (TIGM) generated genetically modified mice using CRISPR**/**Cas9-mediated genome engineering. *Hmga2* single-stranded chimeric guide RNA (sgRNA) 5′TAAGCTGATTAA-3′ were designed as described (Yang *et al.* 2014) and single-stranded DNA donor carrying the mutation was designed with the aim to introduce stop codons in exon 1. Mice with the targeted allele were backcrossed with wild-type (WT) C57BL/6N mice for four generations. Genomic DNA was isolated from the tail biopsies using a DNeasy Blood & Tissue Kits (QIAGEN, USA). Genotyping was carried out using a set of genotyping oligonucleotide primers; HMGA2-gF; 5′-TCGCGGGTGGGCTGAGT-3′ and HMGA2-gR; 5′-TCAGCCCAGGGACAACCT-3′, 107 bp for wild-type and 114 bp for mutant. Total RNA was isolated from whole embryo from day 10 using an RNA isolation kit (Zymogen, USA), and reverse transcription was performed with the SuperScript^™^ IV VILO^™^ Master Mix (Invitrogen, USA). RT-PCR amplifications were performed using a Lightcycler 480 (Bio-Rad, USA). The HMGA2-F; 5′-AGCAAGAGCCAACCTGTGAG-3′ and HMGA2-R; 5′-CCAGCATTTTGTCTCATTCAG-3′ primers were used and PCR products were sequenced to confirm the *Hmga2* mRNA product of the *Hmga2*^−/−^ mice.

### Western blot analysis

Proteins were extracted from 10 days old embryos using the T-PER tissue protein extraction reagent (Thermo Fisher Scientific, USA), and protein concentration determined using the DC protein assay kit (Bio-Rad, USA). A total of 40 μg protein was prepared per sample and electrophoresis was performed on precast 4–20% gel (mini-PROTEIN TGX gel, Bio-Rad, USA) before transfer to Immuno-Blot PVDF membrane (Bio-Rad). After blocking for 1 h with 5% skim milk in 0.1% TBS-Tween, the membrane was incubated overnight at 4°C with 1:1000 dilution of primary antibody, either anti-HMGA2 for C-terminal (sc-130024, Santa Cruz Biotechnology, USA) or N-terminal (#8179, Cell signaling Technology, USA) detection or anti-β-actin (#3700, Cell Signaling Technology, USA). After extensive washing in TBS-T 0.1%, the membrane was incubated for an hour at room temperature with horseradish peroxidase-conjugated secondary antibody and detected with SuperSignal West Femto reagents (Thermo Fisher Scientific, USA) and imaged using ChemiDoc MP (Bio-Rad, USA).

### Skull analysis using micro computed tomography

Micro computed tomography (microCT) was performed on skulls from three *Hmga2*^−/−^ and three wild-type adult males at 14 weeks of age using a Scanco μCT 50 specimen scanner (Scanco Medical AG, Switzerland). After identification of the skull on a scout view radiograph, images were acquired at an isotropic resolution of 6 μm in all three dimensions (integration time of 500 ms; X-ray beam potential 55 kVp; beam intensity 109 μA). For each 180° of imaging, 1000 projections were acquired. Bone was segmented from soft tissue using the same threshold (245 mg HA/cm^3^) for all groups. After scanning, 3D microstructural image data were reconstructed using the Scanco cone-beam reconstruction algorithm (Hildebrand *et al.* 1999). Structural and density calibration of the scanner is routinely performed using a calibration phantom and all analyses are consistent with published American Society for Bone and Mineral Research guidelines for rodents (Bouxsein *et al.* 2010).

Scan DICOM files were imported into 3D Slicer v4.10.2 open-source image analysis software. Rendering was performed using the CT-bones preset, and VTK GPU raycasting adjusted to minimize background signal. Landmarks were placed at the pinnacle of the skull along the coronal suture and the base of the mandible corresponding to the coronal position of the previous landmark to measure skull height; at the tip of the rostrum and the most posterior point of the occipital for skull length; and at the most lateral parts of the skull for width.

### Male fertility assessments

Male breeding trials to assess fertility were set up in trios by pairing one *Hmga2^+/+^* or *Hmga2*^−/−^ males with two wild-type females at least for 2 months and tracking the numbers of litters and pups per litter. Mating behavior was validated by checking for vaginal seminal plugs by 9:00 a.m. the morning after mating. For reproductive tissue and sperm assessments, males were euthanized by CO_2_ asphyxiation and cervical dislocation. Organs collected were weighed and testes and sperm were collected for analyses.

### Testis histology

Testis tissues were fixed in Bouin’s solution for 48 h, then washed in PBS, dehydrated, and paraffin-embedded according to standard histological techniques. Five (5) µm sections were Hematoxylin and Eosin (H&E) stained and imaged on a Leica DMi8 microscope (Leica microsystems, Germany). Tubule diameters were determined using the line measurement tool in the Leica Application Suite X (LAS X) analysis software package. All tubules exhibiting longitudinal sectioning were excluded, while all cross sectioned tubules present in a single testis section per animal were measured and the diameter across the shortest axis recorded to avoid inclusion of transverse sectioned measurements.

### Sperm analysis

Samples of the cauda epididymis were rinsed in PBS, placed into 1 ml Human Tubal Fluid medium (EMD Millipore, USA) and cut with iridectomy scissors to make several incisions and allow sperm to swim out during incubation at 37°C for 15 min. Sperm counts were determined by diluting into diH_2_O and counting using Neubauer chamber slides. The remaining sperm were fixed in 4% PFA for 1 h, then washed with PBS. For morphological assessment, sperm were settled onto Poly-L-lysine coated slides and visualized by differential interference contrast microscopy at 63X or phase-contrast 40X on a Leica DMi8 microscope.

The Click-iT Plus TUNEL assay kit (Invitrogen, USA) was used for terminal deoxynucleotidyl transferase dUTP nick-end-labeling (TUNEL) to assess DNA damage. Incubation, preparation, and staining of PFA fixed sperm and appropriate controls were carried out as per manufacturer’s instructions. Stained samples were settled onto poly-L-lysine coated glass slides, mounted in Prolong Gold Antifade media, and visualized on a Leica DMi8 microscope with Chroma filters for FITC detection. At least three fields of view and 200 sperm were scored for each sample to determine the % of TUNEL-positive sperm per sample.

For CMA3 staining, sperm samples were settled on glass slides. Slides were permeabilized with 0.5% NP40 in PBS for 2 min, rinsed with PBS, then treated for 20 min with 100 μl CMA3 solution: 0.25 mg/mL CMA (Millipore-Sigma, USA) and 10 mM MgCl_2_, McIlvain’s buffer (17 mM citric acid, 164 mM Na_2_HPO_4_, pH 7.0). Slides were rinsed with PBS and mounted with Prolong Gold Antifade Media (Thermo-Fisher, USA). Evaluation of fluorescence was done for a minimum of 200 spermatozoa on each slide.

### Behavioral analyses

#### Open field test

Mice were acclimated for at least 1 h and tested in the open field. The open field consisted of a plastic box divided into four equal sized squares. During testing, animals were placed into the center of the open field, and were videotaped from the top of the arenas in 30 min sessions. Tracking software, Ethovision (Noldus) was used to analyze each parameter. Using the software analysis package, a series of 10 × 10 cm zones was identified and used to evaluate subject tracks. The outer zone consisted of 16 blocks as identified while the inner zone consisted of nine blocks and is shaded. The ethological parameters (defined *thigmotaxis*, when the mouse spent time in the outer zones of the arena, or *central*, when the mouse spent time in the center zones of the arena) comprised of the following behaviors: (1) *rearing*: the mouse reared on its hind paws while in the outer zone of arena, (2) *grooming*: the mouse licked/scratched its fur, washed its face, and/or licked its genitalia, and (3) s*niffing*: the mouse olfactory explored the environment, either motionless or moving.

#### Nest building

The nest building assay was performed as described (Deacon 2006a). Briefly, mice placed into individual test cages with single nesting material for 16 h and nest quality scores were accessed based on the following scores: (1) cotton fiber not noticeably touched (more than 90% intact); (2) cotton fiber partially torn (50–90% remaining intact); (3) cotton fiber mostly shredded but often no identifiable nest site; (4) an identifiable but flat nest; (5) a (near) well-defined nest with walls.

#### Marble burying

The marble burying assay was performed as previously described with minor modifications (Deacon 2006b). Four rows of five marbles were placed on top of unscented bedding material to a depth of 5 cm. Mice were placed in the cage containing marbles for 30 min. Marbles were scored as buried if two-thirds of its surface area was covered by bedding. Experimenters were blind to genotype when tests score were collected.

### Statistical analysis

The JMP pro 14.0.0 software was used for statistical analysis and data were expressed as mean ± SE. Statistical significances between groups or conditions were analyzed by one-way ANOVA by genotype followed by all pair Student’s *t*-test. Differences were considered statistically significant when *P* < 0.05; *P < 0.05, **P < 0.01, and ***P < 0.001. A summary statistics table is included in the Supplementary material as Supplementary Table S1.

## Results

### Generation of *Hmga2^tamu-ko^* mice

**Figure 1:**
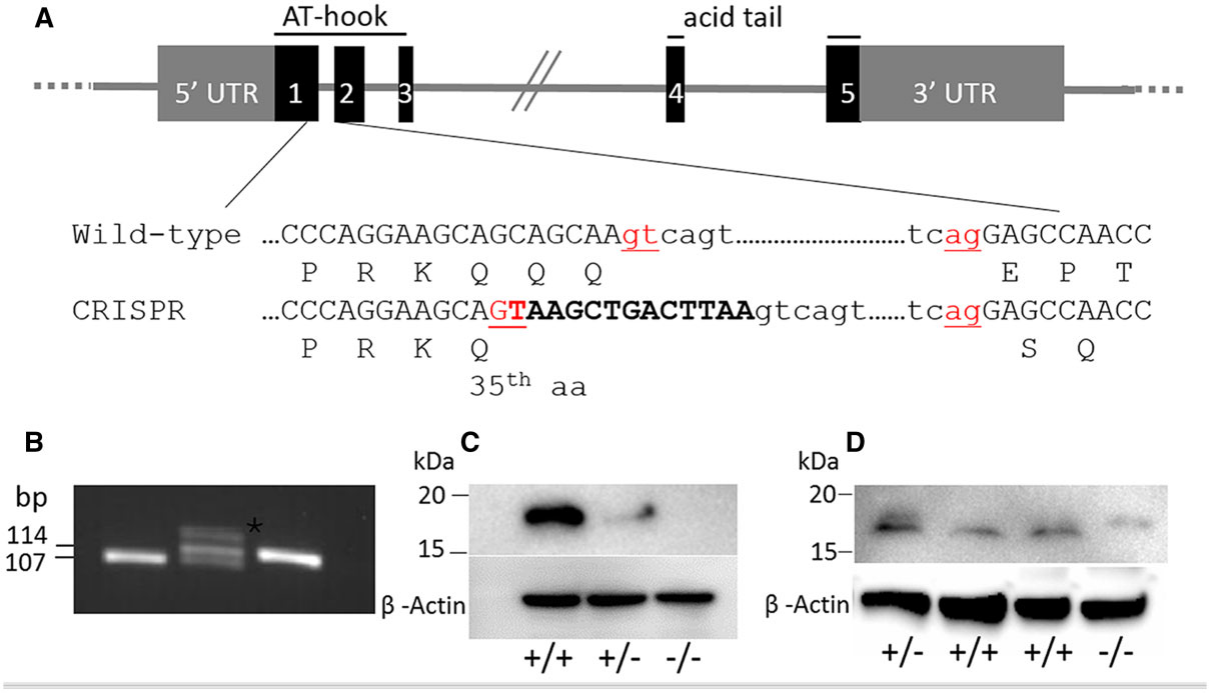
Generation of *Hmga2^tamu-ko^* knockout mice. (A) Schematic illustration of the *Hmga2* knockout strategy. The 13 bp oligomer corresponding to the end of exon 1 is marked in bold and the splicing donor and acceptor sites are shown in red. Amino acids are indicated using the one-letter code. (B) Results of *Hmga2* genotyping assay. A 107 bp wild-type fragment and a 114 bp mutant fragment were generated; heterozygotes showed a heteroduplex band (asterisk) with retarded migration. (C and D) Western blot analysis using antibody that recognizes the C-terminus (C) and the N-terminus (D). ß-actin was used as loading control.

*Hmga2-null* mice were generated using CRISPR/Cas9 methodology. A seven-nucleotide insertion was made to induce a premature stop codon at codon 36 in exon 1 (Figure 1). However, the insertion unexpectedly created a new splice donor site which resulted in a deletion of 14 nucleotides in the *Hmga2* transcript and a frameshift after codon 35 resulting in an alternate open reading frame that could potentially be translated into a protein 72 amino acids longer than the wild-type HMGA2 protein (Figure 1A, Supplementary Figure S1); total predicted length of 180 and 108 amino acids, respectively. The *Hmga2^tamu-ko^* allele was confirmed by PCR followed by sequencing (Figure 1B). Western blot analysis was performed with two different HMGA2 specific antibodies. First, an antibody targeting the C-terminal portion of HMGA2 confirmed the absence of detectable wild-type HMGA2 protein in *Hmga2*^−/−^ mice. As expected, *Hmga2*^+/−^ mice demonstrated the requisite reduction of HMGA2 protein compared to control littermates (Figure 1C). Next, an antibody targeting the most N-terminal region identified HMGA2 proteins of different sizes; wild-type mice express normal HMGA2 while *Hmga2*^−/−^ mice express a protein with a larger size than normal HMGA2 and consistent with a protein composed of 180 amino acids; *Hmga2*^+/−^ express both HMGA2 variants (Figure 1D). Taken together, *Hmga2^tamu-ko^* do not express wild-type HMGA2 but express a variant protein due to the frame shift at the 3’ end of exon 1.

### Effects of *Hmga2* deficiency on growth parameters

We carefully examined genotype segregation ratios and body weight in *Hmga2* mutant mice. *Hmga2*^+/−^ × *Hmga2*^+/−^ mating resulted in genotype proportions that did not deviate significantly from expected Mendelian ratios at birth, with *Hmga2^+/+^*: *Hmga2*^+/−^: *Hmga2*^−/−^ = 1.2:1.8:1.0 (Chi-square = 0.9, *df* = 2; *P=* 0.62) among 61 pups from 6 pregnancies assessed on day 1. However, at 3 weeks of age, the ratios were 1.2:2.2:0.7 (Chi-square = 8.7, *df* = 2; *P=* 0.01) (Supplementary Table S2), indicating that postnatal competition resulted in a deficiency of knock-out animals at 3 weeks of age.

**Figure 2:**
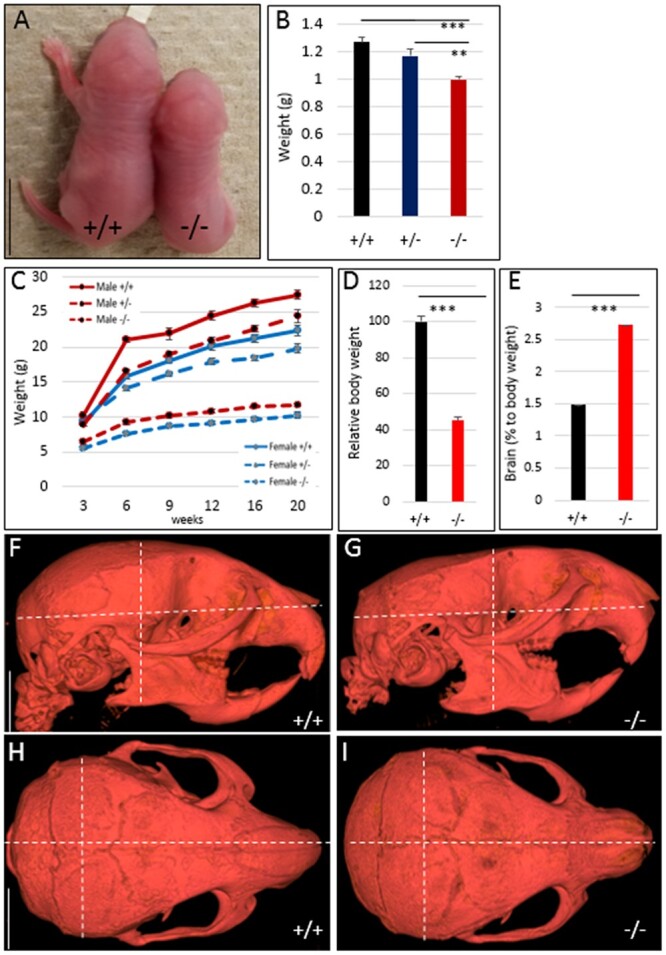
Comparison of body size and growth curves for *Hmga2^+/+^*, *Hmga*2^+/−^, and *Hmga2*^−/−^ mice. (A) Comparison of representative littermates of wild-type and knock-out at day 1. Scale bar = 5 mm. (B) Average body weight for each genotype on day 1, *n* ≥ 5. (C) Growth curves of male and female mice from 3 to 20 weeks of age. *n* = 18/22 *Hmga2^+/+^*, 41/58 *Hmga2*^+/−^, and 24/18 *Hmga2*^−/−^ (female/male). (D and E) Comparison of body weight and brain weights between genotypes at 14 weeks of age. Relative weight loss was determined by dividing gross mass by a control littermate weight, *n* = 4. (F–I) MicroCT analysis for wild-type and knock-out individuals along the sagittal plane (F and G) and frontal plane (H and I). Dotted white lines indicate landmark measurements for length, width, and height that are significantly offset in *Hmga2*^−/−^ animals. Scale bar = 5 mm.

*Hmga2* ^−/−^ mice showed a significantly reduced body size at day 1 compared to littermates (Figure 2, A and B), and this growth retardation was retained throughout the entire life span (Figure 2C). In adult mice, disruption of one *Hmga2* allele (*Hmga2*^+/−^) resulted in an approximately 20% reduction in body weight, while the reduction was about 60% in *Hmga2*^−/−^ mice (Figure 2, C and D). This reduction has been documented previously across most organs (Benson and Chada 1994), with the notable exception of brain, which shows about twofold increase in *Hmga2*^−/−^ mice relative to body size (Figure 2E). As a result, *Hmga2*^−/−^ homozygotes have a disproportionately larger head compared to the rest of the body consistent with the phenotypic effects of *HMGA2* inactivation in rabbits (Carneiro *et al.* 2017).

To further investigate the craniofacial defects of *Hmga2*^−/−^ mice, we utilized microCT. Measurements of skull length, width and height, and surface volume using microCT of *Hmga2*^−/−^ and wild-type adult male mice demonstrated that there is a 15% reduction in skull height and width in concert with similar reduction in brain mass. There is an allometric reduction in skull length in knock-out mice (Figure 2, F–I, Table 1 and Supplementary Figure S2), but no specific reduction of skull length as noted in dwarf rabbits which are heterozygous for a 12.1 kb deletion overlapping the promoter region and the first three exons of *HMGA2* (Carneiro *et al.* 2017).

**Table 1:** Measurements of length, height, and width of the skull for three *Hmga2^tamu-ko^* and three wild-type mice at 14 weeks of age based on microCT scans

	Length (mm)	Height (mm)	Width (mm)	Length/height ratio	Length/width ratio	Surface area (mm^2^)
Knock-out 1	18.3	9.4	9.6	1.95	1.91	1594.0
Knock-out 2	19.0	9.2	10.0	2.07	1.90	1783.3
Knock-out 3	19.1	9.4	11.2	2.03	1.71	1655.7
Wild-type 1	22.3	10.5	10.4	2.12	2.14	2332.5
Wild-type 2	21.7	11.1	10.5	1.95	2.07	2227.9
Wild-type 3	21.2	10.5	11.2	2.02	1.89	2133.2

### Effects of *Hmga2* inactivation on male reproduction

**Figure 3:**
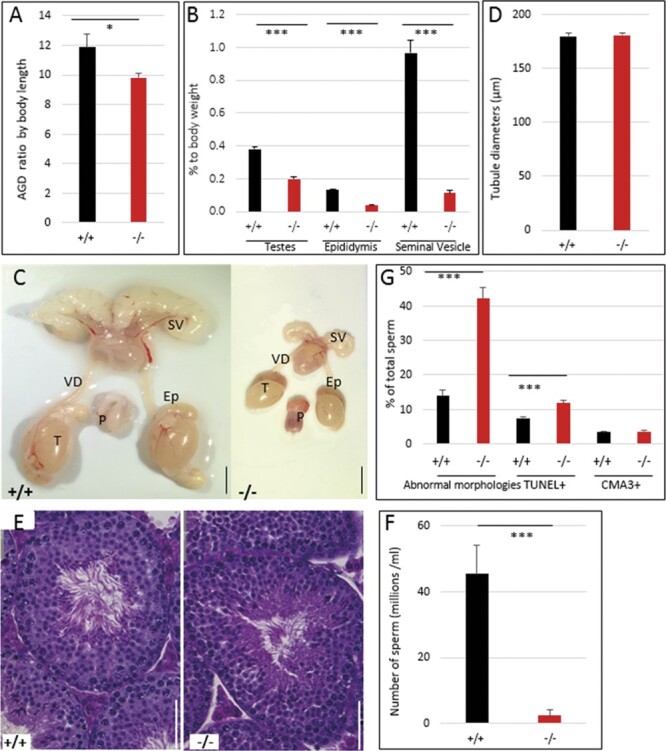
Significant impairment of male reproductive organs in *Hmga2^tamu-ko^* mice. (A) AGD corrected for body length. (B) Relative reduction of reproductive organs. (C) Representative images of the genital track of *Hmga2^+/+^* (left) and *Hmga2*^−/−^ (right) male mice at 14 weeks of age. Scale bar = 5 mm. (D) Tubule diameters were determined in *Hmga2^+/+^* and *Hmga2*^−/−^ male mice. (E) Representative hematoxylin and eosin-stained sections of testis from *Hmga2^+/+^* (left) and *Hmga2*^−/−^ (right) mice. Scale bar = 50 μm. (F) Total epididymal sperm counts from 14 weeks old males (*n* = 6). (G) Percent of sperms with abnormal morphologies, DNA damage (TUNEL+) and CMA3-stained cells from wild-type and knock-out mouse testes. Ep, epididymis; P, penis; SV, seminal vesicle; T, testis; VD, vas deferens.

Both male and female *Hmga2*^−/−^ mice were found to be sterile, whereas heterozygotes showed reproductive characteristics similar to wild-type mice (Supplementary Table S3) consistent with previous reports for *Hmga2^pg^* genotypes (King 1950). In the *Hmga2^pg^* model, cryptorchidism was also noted. In this study, cryptorchidism was not detected, and testes were observed to transit through the inguinal canal and localize in the scrotum normally. However, it was noted that the anal-genital distance (AGD) was significantly shorter in *Hmga2*^−/−^ mice than in wild-type males, even when corrected for differences in total body length (Figure 3A). In addition, 70% of knock-out males possessed an externalized/prolapsed penis phenotype (Supplementary Figure S3, A–C). To determine if male infertility was due to mating behavior deficits, *Hmga2*^−/−^ males were paired with wild-type females and females were observed for the presence of vaginal plugs as confirmation of copulation. During 2 weeks of observation, three out of seven *Hmga2*^−/−^ males with the prolapse penile phenotype, and three out of four wild-type males produced copulatory plugs indicating that *Hmga2*^−/−^ males do copulate, even when they exhibit penile prolapse.

Compared to wild-type, reproductive tissues of *Hmga2*^−/−^ males showed a reduction of weight relative to body weight, especially seminal vesicles with about 90% reduction (Figure 3, B and C). Despite the significant reduction in the mass of testis, epididymis, and seminal vesicle in *Hmga2*^−/−^ males, testis histomorphology at 14 weeks of age appeared normal, including seminiferous tubule diameters (Figure 3D) and normally organized seminiferous epithelium exhibiting complete spermatogenesis (Figure 3E). *Hmga2*^−/−^ mice exhibited a 10-fold reduction in epididymal sperm count (Figure 3F). The analysis of sperm morphology revealed an increase in morphological abnormalities affecting for instance sperm head shape and flagella wrapping (Figure 3G and Supplementary Figure S3, D–K). Furthermore, TUNEL staining revealed increased sperm DNA damage associated with cell death, whereas sperm chromatin condensation appeared to be unaffected as the number of sperms staining with Chromomycin A3 was similar in wild-type and knock-out males (Figure 3G).

### Behavioral changes in *Hmga2^tamu-ko^* mice

**Figure 4:**
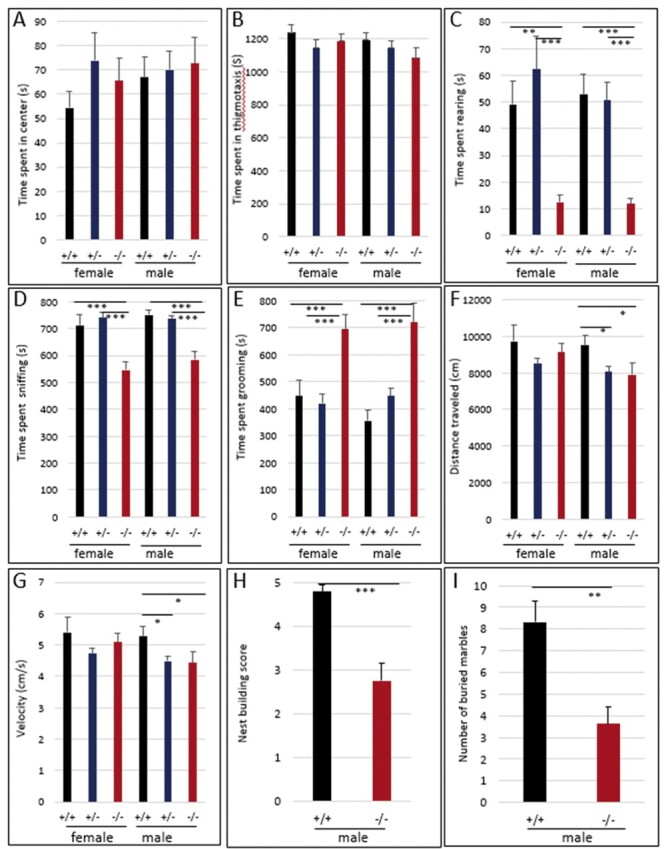
Altered behavior in *Hmga2*^−/−^ mice. *Hmga2^+/+^* (*n* = 44), *Hmga2*^+/−^ (*n* = 54), and *Hmga2*^−/−^ (*n* = 40) mice were subjected to an open field and time spent (A) rearing on the hind paws, (B) sniffing, and (C) grooming were measured. Total distance traveled (D) and velocity (E) were also analyzed. (F) Nest building was assessed by giving mice nesting materials and nest quality was subsequently scored. *Hmga2^+/+^*: *n* = 9 and *Hmga2*^−/−^: *n* = 12. (G) Two observers, blind to the genotype of tested mice, scored male mice for the number of marbles buried. *Hmga2^+/+^*: *n* = 9 and *Hmga2*^−/−^: *n* = 12.

In humans, some cases with the 12q14 microdeletion syndrome show autism and other behavioral difficulties (Lynch *et al.* 2011; Dória *et al.* 2020). Thus, in an attempt to study if *Hmga2* inactivation in mice affects behavior, a 30-min open-field test was performed to evaluate exploratory and anxiety/stress‐linked behavior in the mouse model. No significant differences were observed among genotypes for the time and frequencies spent in center and in thigmotaxis (Figure 4, A and B). However, the time spent in exploratory behavior such as rearing up on the hind paws and sniffing were significantly reduced in both *Hmga2*^−/−^ males and females (Figure 4, C and D). Conversely, *Hmga2*^−/−^ mice showed a marked increase in time spent grooming the face and body (Figure 4E). Significantly reduced velocity and total distance moved during the open field test were also detected in male *Hmga2*^−/−^ mice (Figure 4, F and G). As a result, additional behavioral investigations focused solely on males. Significantly reduced scores were observed for male *Hmga2*^−/−^ mice in a marble burying test and in nest building behavior compared to wild-type controls (Figure 4, H and I). The results indicated that the loss of *Hmga2* significantly affected exploratory, marble burying, and nest-building behaviors.

## Discussion

The original observation of a 40–50% reduction in body weight for *Hmga2^pg^* and *Hmga2p^pg-TgS40ACha^* homozygotes (King 1950; Benson and Chada 1994) was confirmed by the 60% reduction observed using the *Hmga2^tamu-ko^* model described here. The disproportionately larger brain of *Hmga2^tamu-ko^* homozygotes is consistent with the phenotypic characterization of *Hmga2^pg-TgS40ACha^* mice (Benson and Chada 1994). In the brain, *Hmga2* expression was weak and limited to the cerebellum and forebrain during development unlike other regions of the body (Zhou *et al.* 1995; Hirning-Folz *et al.* 1998). This provides a possible explanation for why the inactivation of *Hmga2* induces a significant reduction in body weight but only modest reductions in brain volume.

*HMGA2* ^−/−^ rabbits die soon after birth (Carneiro *et al.* 2017) and human *HMGA2* null alleles are presumably recessive lethal since a null homozygote has never been reported. In contrast, *Hmga2 ^pg-TgS40ACha^* mice homozygotes have a normal life span and occur at the expected Mendelian ratio (Benson and Chada 1994). In the *Hmga2^tamu-ko^* model, *Hmga2*^−/−^ mice are born at the expected Mendelian ratios at birth, however, 40% of the homozygous mutants are lost before 3 weeks of age. Thus, reduced body size maybe disadvantageous when competing for limited resources, or alternatively, undefined developmental defects may cause a higher postnatal mortality.

The altered craniofacial phenotype (brachycephaly) in *HMGA2*-null rabbits (Carneiro *et al.* 2017) along with human GWAS indicating an association with craniofacial morphology, especially in the upper region of the face and nose (Fatemifar *et al.* 2013; Moreno Uribe *et al.* 2015), suggests that *HMGA2* may have possible pleiotropic effects on craniofacial development. The *Hmga2*^−/−^ mice generated in this study showed only a 15% reduction in cranial size compared to the 60% reduction in total body weight. Though there are subtle differences in skull shape there is no indication of nonallometric change suggesting no specific effect on craniofacial development. The 12q14 microdeletion syndrome in humans, involving the loss of several genes including *HMGA2*, shows growth delay in the womb, leading to short stature and occasional macrocephaly (Takenouchi *et al.* 2012). In cases where *HMGA2* was not affected by the deletion, growth was within the normal centile, while other cases with a deletion only disrupting *HMGA2* showed short stature but did not have macrocephaly (Ligon *et al.* 2005; Lynch *et al.* 2011; Takenouchi *et al.* 2012). These results in humans and mice strongly suggest that *HMGA2* has a major effect on growth and body stature but no specific effect on craniofacial development. The observed association between *HMGA2* and craniofacial phenotypes based on GWAS studies in humans (Moreno Uribe *et al.* 2015) and in Darwin’s finches (Lamichhaney *et al.* 2016) as well as the deletion causing the dwarf phenotype in rabbits (Carneiro *et al.* 2017) maybe caused by altered expression of closely linked genes, in particular if there are regulatory elements affecting these genes within or in the near vicinity of *HMGA2*.

In mice, *Hmga2^pg^* and *In(10)17Rk* variants were presumed to be sterile due to abnormal spermatogenesis and cryptorchidism, as well as priapism at 6 weeks of age (King 1950; Chieffi *et al.* 2002). In pig, targeted *HMGA2* null males exhibited impaired fertility with evidence of impaired spermatogenesis, and cryptorchidism (Chung *et al.* 2018). Our study also documented male sterility, reduced testis size, and about 70% of knock-out males displayed penile prolapse by the age of 3 weeks. However, our mice did not exhibit cryptorchidism and morphological and histological observation of the knock-out testes showed grossly normal spermatogenesis contrary to previous reports of complete azoospermia associated with homozygosity for *In(10)17Rk* (Chieffi *et al.* 2002). It has been proposed that the HMGA2 protein may be involved in chromatin condensation during G2/M transition of mouse spermatocytes by interacting with NEK2, which phosphorylates HMGA2 in a MAPK-dependent manner and release HMGA2 from chromatin (Di Agostino *et al.* 2004). Furthermore, deletion of *Hmgb2*, encoding another member of the HMG protein family, showed spermatogenesis defects with subfertility suggesting a role in germ cell differentiation (Ronfani *et al.* 2001). Taken together, the data suggest a role for HMG proteins in male fertility where HMGB2 is required for spermatogonia function, while our data indicate that HMGA2 is required for normal-sized testis and sperm viability. It is well understood that Sertoli cell number is a key regulator of testis size and spermatogenic potential (Rebourcet *et al.* 2017). Our data are consistent with such a potential role for HMGA2.

This is the first study indicating behavioral consequences of *Hmga2* loss-of-function. The open field test demonstrated that *Hmga2*^−/−^ mice spent more time grooming and showed a significant decrease in exploratory behavior (rearing and sniffing, Figure 4). This may suggest an increase of repetitive behavior but that was not supported because the *Hmga2*^−/−^ mice exhibited reduced marble burying and nest-building behaviors. However, increased grooming does not only represent repetitive behavior but is also considered to reflect emotionality including stress-induced behavior and is inversely related to exploration (Mu *et al.* 2020), suggesting that *Hmga2*^−/−^ mice may experience high levels of emotionality which induced the decrease in exploratory behavior. One possible explanation of emotionality may be related to the dramatic reduction of body size due to *Hmga2* inactivation which could be a negative stressor as the knockout mice have to compete with normal-sized littermates for food and they in fact show a 40% increase of postnatal mortality (Supplementary Table S2). Thus, it is an open question whether our observed effects on behavior is caused by a direct effect of HMGA2 on brain function or an indirect effect due to the effect of HMGA2 on body size, or a combination of these possibilities. A direct effect on brain development is definitely a possibility as it for instance has been reported in mouse that regulation of *Hmga1* and *Hmga2* expression are important for the timings of neurogenesis in neocortex by inducing open chromatin state (Kishi *et al.* 2012; Kageyama *et al.* 2019). Human HMGA2 is also known to regulate proliferation of neuron progenitor cell in hypothalamus, central regulator of behavior and vital physiological homeostasis (Zhou *et al.* 2020). However, at present we cannot formally exclude the possibility that the effect on behavior is caused by the expression of the variant protein generated due to the frame shift in the targeted allele. It will therefore be important to confirm the effect on behavior using other *Hmga2* variants in mice, such as the pygmy allele caused by a large deletion of the 5′ end of the gene.

In summary, the studies described herein demonstrate the importance of *Hmga2* for controlling growth, normal reproduction and behavior. Using CRISPR/Cas9 a *Hmga2* coding region-specific null allele *Hmga2^tamu-ko^* was developed. Detailed analyses revealed that inactivation of *Hmga2* resulted in a complex phenotype with growth retardation in prenatal and postnatal development, penile prolapse, sterility in both males and females, and altered behavior.

## Data availability

Strain are available upon request. The authors affirm that all data necessary for confirming the conclusions of the article are present within the article, figures, and tables.

Supplementary material is available at *G3* online.

## Supplementary Material

jkab417_Supplementary_Table_S1

jkab417_Supplementary_Table_S2

jkab417_Supplementary_Table_S3

jkab417_Supplementary_Figure_S1

jkab417_Supplementary_Figure_S2

jkab417_Supplementary_Figure_S3
