# Supplementary material for: Hmga2 deficiency is associated with allometric growth retardation, infertility, and behavioral abnormalities in mice
Source: G3 (Bethesda). 2021 Dec 8;12(2):jkab417. doi: 10.1093/g3journal/jkab417 (PMC9210324; doi:10.1093/g3journal/jkab417)
Supplement: jkab417_Supplementary_Table_S2 [file jkab417_supplementary_table_s2.docx]

Table S2. Segregation of the *Hmga2^tamu-KO^* allele in +/- x +/- matings

| Age | Genotype distribution | | | Chi-square  (d.f.=2) | P-value |
| --- | --- | --- | --- | --- | --- |
|  | +/+ | +/- | -/- |  |  |
| Day 1 | 18 | 27 | 16 | 0.93 | 0.63 |
| Week 3 | 74 | 139 | 44 | 8.71 | 0.01 |
