# Supplementary material for: Hmga2 deficiency is associated with allometric growth retardation, infertility, and behavioral abnormalities in mice
Source: G3 (Bethesda). 2021 Dec 8;12(2):jkab417. doi: 10.1093/g3journal/jkab417 (PMC9210324; doi:10.1093/g3journal/jkab417)
Supplement: jkab417_Supplementary_Table_S3 [file jkab417_supplementary_table_s3.docx]

Table S3. Fertility assessments in matings between different combinations of *Hmga2* genotypes

| Mating | | Cumulate time in mating | # of pair | # of pregnancies |
| --- | --- | --- | --- | --- |
| male | female | (months) |  |  |
| *Hmga2*^-/-^ | *Hmga2*^+/+^ | 28 | 10 | 0 |
| *Hmga2* ^+/+^ | *Hmga2*^-/-^ | 33 | 10 | 0 |
| *Hmga2*^+/-^ | *Hmga2*^+/-^ | 20 | 10 | 31 |
| *Hmga2*^+/+^ | *Hmga2*^+/+^ | 24 | 10 | 33 |
