# Supplementary material for: Hmga2 deficiency is associated with allometric growth retardation, infertility, and behavioral abnormalities in mice
Source: G3 (Bethesda). 2021 Dec 8;12(2):jkab417. doi: 10.1093/g3journal/jkab417 (PMC9210324; doi:10.1093/g3journal/jkab417)
Supplement: jkab417_Supplementary_Figure_S1 [file jkab417_supplementary_figure_s1.pdf]

Hmga2$^{+/+}$	MSARGEGAGQPSTSAQGQPAAPVPQ**KRGRGRPRK**QQQEPTCEPSP**KRPRGRPK**GSKNKSP	60
Hmga2$^{-/-}$	MSARGEGAGQPSTSAQGQPAAPVPQ**KRGRGRPRK**QSQPVSPLLRDPEEDPKAAKTRAPLK	60
	*********************************** *	
Hmga2$^{+/+}$	SKAAQKKAETIGE**KRPRGRPRK**WPQQVVQKKPAQETEETSSQESAEED	108
Hmga2$^{-/-}$	QPRRKQRPLEKNGQEADLGNGHNKSFRRSLLRRLKRHPRKSPQRRIRGRRHSISTSASVG	120
Hmga2$^{-/-}$	SFEGRRHCSDQLFLTATVFLLPAGWGGGGAGRGAGPGWAKSHNLEKDYINHFVIPSQSQV	180

Figure S1. Amino acid alignment of HMGA2 sequences. Three conserved DNA binding domains in bold, the invariant repeat (R-G-R-P) are in grey boxes and the amino acid residues that differ between wild-type and knockout alleles are underlined. An asterisk indicates sequence identity. Yellow indicates the first amino acid altered in the *Hmga2*$^{tamu\text{-}ko}$ allele due to alternative splicing.
