# Supplementary material for: Hmga2 deficiency is associated with allometric growth retardation, infertility, and behavioral abnormalities in mice
Source: G3 (Bethesda). 2021 Dec 8;12(2):jkab417. doi: 10.1093/g3journal/jkab417 (PMC9210324; doi:10.1093/g3journal/jkab417)
Supplement: jkab417_Supplementary_Figure_S2 [file jkab417_supplementary_figure_s2.pdf]

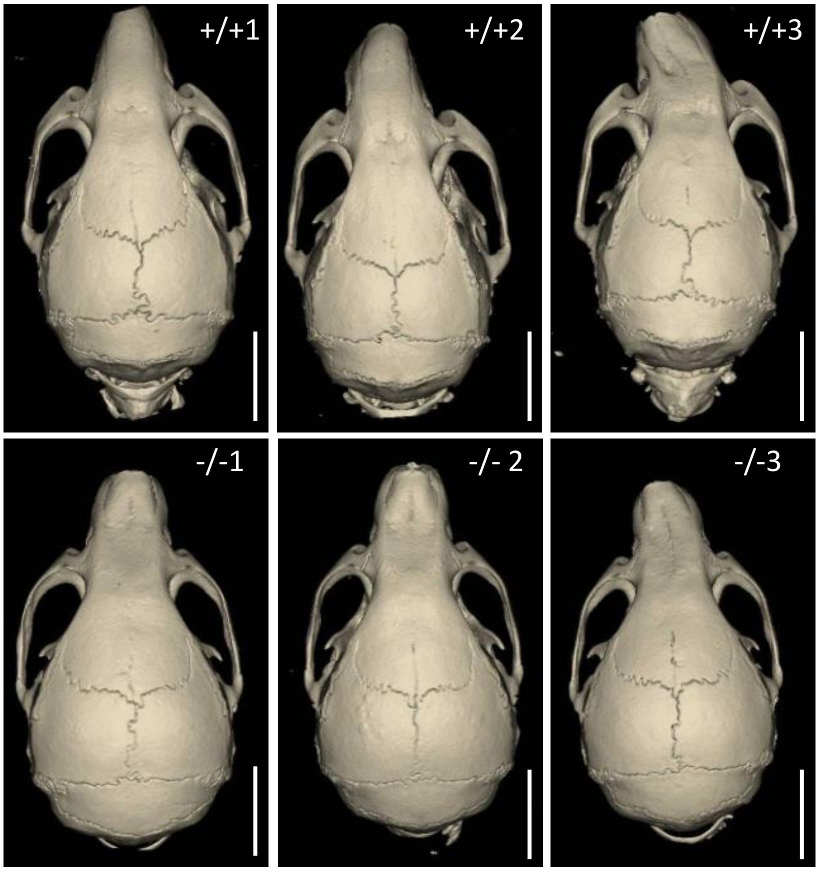

Figure S2. MicroCT image for three *Hmga2*$^{+/+}$ and *Hmga2*$^{-/-}$ mice at 14 weeks of age along the frontal plane. +/+: *Hmga2*$^{+/+}$ and -/-: *Hmga2*$^{-/-}$ Unit bar scale=5mm
