# Supplementary material for: Hmga2 deficiency is associated with allometric growth retardation, infertility, and behavioral abnormalities in mice
Source: G3 (Bethesda). 2021 Dec 8;12(2):jkab417. doi: 10.1093/g3journal/jkab417 (PMC9210324; doi:10.1093/g3journal/jkab417)
Supplement: jkab417_Supplementary_Figure_S3 [file jkab417_supplementary_figure_s3.pdf]

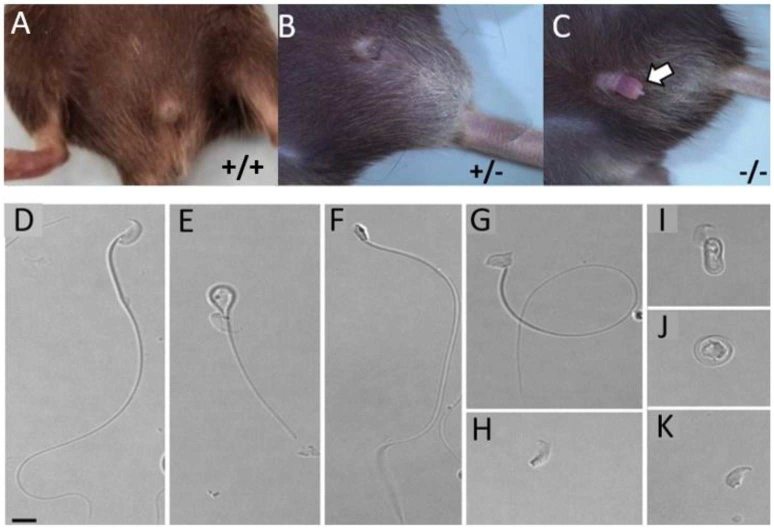

Figure S3. Representative images of the external reproductive organ of *Hmga2*$^{+/+}$, *Hmga2*$^{+/-}$, and *Hmga2*$^{-/-}$ male mice at 14 weeks of age. (A) *Hmga2*$^{+/+}$, (B) *Hmga2*$^{+/-}$ and (C) *Hmga2*$^{-/-}$ male mice. Externalized/prolapse penis of *Hmga2*$^{-/-}$ mice is indicated by an arrow. Representative images of sperm scored (D) as normal (E-K) or abnormal in *Hmga2*$^{+/+}$ and *Hmga2*$^{-/-}$ mice.
